# Impacts of CA9 Gene Polymorphisms and Environmental Factors on Oral-Cancer Susceptibility and Clinicopathologic Characteristics in Taiwan

**DOI:** 10.1371/journal.pone.0051051

**Published:** 2012-12-04

**Authors:** Ming-Hsien Chien, Jia-Sin Yang, Yin-Hung Chu, Chien-Huang Lin, Lin-Hung Wei, Shun-Fa Yang, Chiao-Wen Lin

**Affiliations:** 1 Wan Fan Hospital, Taipei Medical University, Taipei, Taiwan; 2 Graduate Institute of Clinical Medicine, College of Medicine, Taipei Medical University, Taipei, Taiwan; 3 Institute of Medicine, Chung Shan Medical University, Taichung, Taiwan; 4 Graduate Institute of Medical Sciences, College of Medicine, Taipei Medical University, Taipei, Taiwan; 5 Department of Oncology, National Taiwan University Hospital, Taipei, Taiwan; 6 Department of Medical Research, Chung Shan Medical University Hospital, Taichung, Taiwan; 7 Institute of Oral Sciences, Chung Shan Medical University, Taichung, Taiwan; 8 Department of Dentistry, Chung Shan Medical University Hospital, Taichung, Taiwan; Memorial University of Newfoundland, Canada

## Abstract

**Background:**

In Taiwan, oral cancer has causally been associated with environmental carcinogens. Carbonic anhydrase 9 (CA9) is reportedly overexpressed in several types of carcinomas and is generally considered a marker of malignancy. The current study explored the combined effect of *CA9* gene polymorphisms and exposure to environmental carcinogens on the susceptibility of developing oral squamous cell carcinoma (OSCC) and the clinicopathological characteristics of the tumors.

**Methodology and Principal Findings:**

Four single-nucleotide polymorphisms (SNPs) of the *CA9* gene from 462 patients with oral cancer and 519 non-cancer controls were analyzed by a real-time polymerase chain reaction (PCR). While the studied SNPs (*CA9* rs2071676, rs3829078, rs1048638 and +376 Del) were not associated with susceptibility to oral cancer, the GAA haplotype of 3 *CA9* SNPs (rs2071676, rs3829078, and rs1048638) was related to a higher risk of oral cancer. Moreover, the four *CA9* SNPs combined with betel quid chewing and/or tobacco consumption could robustly elevate susceptibility to oral cancer. Finally, patients with oral cancer who had at least one G allele of *CA9* rs2071676 were at higher risk for developing lymph-node metastasis (p* = *0.022), compared to those patients homozygous for AA.

**Conclusions:**

Our results suggest that the haplotype of rs2071676, rs3829078, and rs1048638 combined has potential predictive significance in oral carcinogenesis. Gene-environment interactions of *CA9* polymorphisms, smoking, and betel-quid chewing might alter oral cancer susceptibility and metastasis.

## Introduction

Oral cancers can originate in any tissues of the mouth, but approximately 90% are squamous cell carcinomas (SCCs). Such cancers are known worldwide for their poor prognosis and major oncologic problems [1]. The susceptibility of an individual to oral cancer is mediated by genetic factors and carcinogen-exposure behaviors [2], [3]. Among genetic factors, single-nucleotide polymorphisms (SNPs) are the most common type of DNA sequence variation which influence the occurrence and progression of gene-related diseases. An SNP is a variation in the DNA sequence that occurs when a nucleotide (A, T, C, or G) is changed in at least 1% of a certain population. When an SNP falls in a coding sequence, it may determine a change of an amino acid in the related protein sequence. Such an SNP is called nonsynonymous. In accordance with the degeneracy rules of the genetic code, an SNP can also generate the same amino acid, which is then called a synonymous SNP. Of note, several groups indicated that an SNP in a noncoding region (3′-untranslated region; UTR) of a gene may also impact biological processes [4].

Cancer is a gene-defect disease, and SNPs may possibly predict the risk of oral cancer [5]. Hence, strategies of genotyping related SNPs and analyzing their distribution frequencies in a community are often used to predict the risk and prognosis of cancers. Betel-quid chewing, tobacco use, and alcohol consumption are 3 common carcinogen-exposure behaviors. Combinations of these environmental carcinogens and certain gene polymorphisms might increase a person’s susceptibility to oral cancer [5]. Thus, SNPs in certain genes might affect one’s response to stimulation toward carcinogenesis promoted by environmental factors.

Hypoxia is a common feature of human solid tumors [6]. Tumor hypoxia causes tumor cells to undergo adaptive changes that enable them to survive and proliferate [7]. Hypoxic tumors are associated with aggressive tumor growth, metastasis, and treatment failure in several types of solid human tumors [8]. The development of a marker for hypoxic tumors could allow assessment of the biologic aggressiveness of individual tumors, which could, in turn, facilitate individually tailored treatments.

Carbonic anhydrase 9 (CA9), a glycoprotein belonging to a family of zinc-containing enzymes, is not expressed in most organs or tissues, but it is abundantly expressed in numerous cancers and has been investigated as an endogenous marker for tumor hypoxia [9], [10]. *CA9* is located on chromosome 9p12–13, which comprises 11 exons and encodes for the 459-amino-acid protein, CA9. CA9 is a membrane-associated protein that catalyzes the reversible reaction H_2_O+CO_2_↔H^+^+HCO_3_^−^, which is crucial to a wide variety of processes, including pH regulation [11]. Through this activity, CA9 helps maintain a normal pH in tumor cells in a hypoxic microenvironment, which may allow tumor cell proliferation [12]. Previous studies demonstrated that CA9 expression represents biologic tumor aggressiveness and is associated with poor clinical outcomes in several tumors including head and neck, cervix, kidney, and lung cancers [13]–[16]. More recently, aberrant expression of CA9 in OSCC was also demonstrated to be correlated with nodal metastasis and a poor prognosis [17]–[19]. These various findings suggest that CA9 plays an important role in OSCC progression.

**Figure 1:**
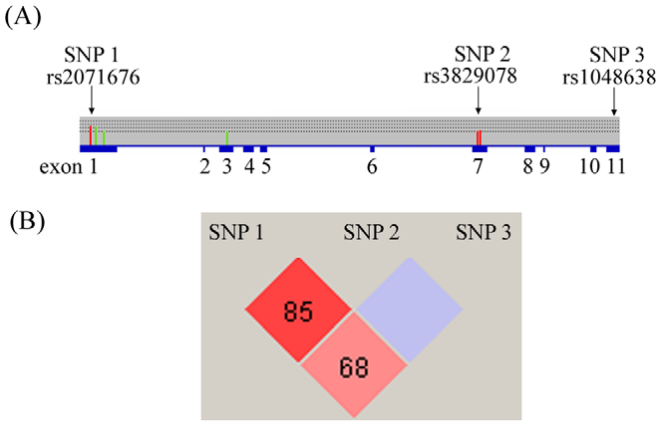
Linkage disequilibrium (LD) and haplotype block structure of the *CA9* gene. Numbers in the diamonds represent the pair-wise *D’* values. This plot was generated by the Haploview program.

Prior research reported that polymorphic variations in the exon region of *CA9* were associated with overall survival for metastatic renal cell carcinoma [11]. Apart from that study, no reports focused on the association between *CA9* polymorphisms and the development of solid tumors. The current study investigated relationships between SNPs (rs2071676, rs3829078, and 376del393) in the exon and 3′-UTR (rs1048638) regions of the *CA9* gene and the risk of oral cancer (Figure 1). The influences of these SNPs combined with betel-nut and tobacco consumption, leading to a susceptibility to oral cancer, were evaluated. We also investigated the relationship between genetic influences and the clinicopathological characteristics of oral cancer. To our knowledge, this is the first study to demonstrate a significant association between *CA9* polymorphisms and oral carcinogenesis in Taiwan.

## Results

The statistical analysis of demographic characteristics is shown in Table 1. We found significantly different distributions of age (*p*  = 0.023), gender (*p*<0.001), betel-quid chewing (*p*<0.001), alcohol consumption (*p*<0.001), and tobacco use (*p*<0.001) between control participants and OSCC patients. To diminish the possible interference of environmental factors, the AORs with 95% CIs were estimated by multiple logistic regression models after controlling for other covariates in each comparison.

**Table 1:** Distributions of demographic characteristics in 519 healthy controls and 462 patients with oral cancer.

Variable	Controls (*N* = 519)	Patients (*N* = 462)	*p* value
Age (yr)	Mean ± S.D.	Mean ± S.D.	
	52.43±14.67	54.35±11.40	*p* = 0.023*
Gender	*n* (%)	*n* (%)	
Male	425 (81.9%)	444 (96.1%)	
Female	94 (18.1%)	18 (3.9%)	*p*<0.001*
Betel-nut chewing			
No	428 (82.5%)	109 (23.6%)	
Yes	91 (17.5%)	353 (76.4%)	*p*<0.001*
Alcohol consumption			
No	308 (59.3%)	177 (38.3%)	
Yes	211 (40.7%)	285 (61.7%)	*p*<0.001*
Tobacco consumption			
No	309 (59.5%)	74 (16.0%)	
Yes	210 (40.5%)	388 (84.0%)	*p*<0.001*

The Mann-Whitney *U*-test or Fisher’s exact test was used between healthy controls and patients with oral cancer. * Statistically significant at *p*<0.05.

All analyzed gene markers in our control group were statistically verified as being in HWE (*p*>0.05). Data in Table 2 show that for both OSCC patients and controls, alleles with the highest distribution frequency were as follows: heterozygous A/G for the +201 (rs2071676) locus and homozygous for A/A, C/C, and Ins/Ins, respectively, for the +1081 (rs3829078), +1584 (rs1048638), and +376 (376del393) loci of the *CA9* gene. After adjusting for the variables, there was no significant difference in having oral cancer in individuals with the rs2071676, rs3829078, rs1048638, and 376del393 polymorphisms of *CA9* gene compared to wild-type (WT) individuals.

**Table 2:** Distribution frequency of *CA9* genotypes in 519 healthy controls and 462 oral-cancer patients.

Variable	Controls (*N* = 519) *n* (%)	Patients (*N* = 462) *n* (%)	OR (95% CI)	AOR (95% CI)
**rs2071676**				
AA	144 (27.7%)	116 (25.1%)	1.00	1.00
AG	262 (50.5%)	238 (51.5%)	1.128 (0.835–1.524)	1.240 (0.806–1.908)
GG	113 (21.8%)	108 (23.4%)	1.186 (0.828–1.700)	1.056 (0.634–1.759)
AG+GG	375 (72.3%)	346 (74.9%)	1.145 (0.861–1.523)	1.179 (0.785–1.772)
**rs3829078**				
AA	484 (93.3%)	417 (90.3%)	1.00	1.00
AG	35 (6.7%)	45 (9.7%)	1.492 (0.941–2.366)	0.901 (0.470–1.726)
GG	0 (0%)	0 (0%)	**–**	**–**
AG+GG	35 (6.7%)	45 (9.7%)	1.492 (0.941–2.366)	0.901 (0.470–1.726)
**rs1048638**				
CC	455 (87.7%)	404 (87.4%)	1.00	1.00
CA	61 (11.8%)	54 (11.7%)	0.997 (0.675–1.472)	1.197 (0.682–2.102)
AA	3 (0.5%)	4 (0.9%)	1.502 (0.334–6.750)	2.755 (0.389–19.513)
CA+AA	64 (12.3%)	58 (12.6%)	1.021 (0.698–1.492)	1.267 (0.734–2.187)
**376del393**				
INS/INS	394 (75.9%)	370 (74.2%)	1.00	1.00
INS/Del	120 (23.1%)	114 (23.2%)	1.024 (0.760–1.380)	1.081 (0.710–1.645)
Del/Del	5 (1.0%)	5 (2.6%)	2.757 (0.962–7.904)	2.080 (0.551–7.853)
INS/Del+ Del/Del	125 (24.1%)	119 (25.8%)	1.094 (0.818–1.461)	1.135 (0.756–1.706)

The odds ratios (ORs) and with their 95% confidence intervals (CIs) were estimated by logistic regression models. The adjusted ORs (AORs) with their 95% CIs were estimated by multiple logistic regression models after controlling for age, gender, betel-nut chewing, and tobacco and alcohol consumption.

Interaction effects between environmental risk factors and genetic polymorphisms of *CA9* are shown in Tables 3 and 4. Among 598 smokers, subjects with at least 1 G allele of rs2071676, 1 G allele of rs3829078, 1 A allele of rs1048638, or 1 Del allele of *CA9*+376 and the betel-nut-chewing habit had respective risks of 23.90–(95% CI: 8.84–64.61), 14.25–(95% CI: 5.18–39.17), 16.80–(95% CI: 5.82–48.46), and 12.85-fold (95% CI: 6.19–26.68) of having oral cancer. Individuals with either at least 1 G allele of rs2071676, 1 G allele of rs3829078, 1 A allele of rs1048638, or one Del allele of *CA9*+376 or who chewed betel nut had respective risks of 4.36- (95% CI: 1.67–11.42), 15.18–(95% CI: 8.92–25.83), 10.65–(95% CI: 6.39–17.77), and 11.30-fold (95% CI: 6.50–19.64) of having oral cancer compared to individuals with WT homozygotes who did not chew betel nut (Table 3).

**Table 3:** Adjusted odds ratios (ORs) (AORs) and 95% confidence intervals (CIs) of oral cancer associated with *CA9* genotypic frequencies and betel-nut chewing among 598 smokers.

Variable	Controls (*n* = 210) (%)	Patients (*n* = 388) (%)	OR (95% CI)	AOR (95% CI)
**rs2071676**				
aAA genotype and no betel-nut chewing	32 (15.2%)	8 (2.1%)	1.00	1.00
bAG or GG genotype or betel-nut chewing	127 (60.5%)	141 (36.3%)	4.441 (1.974–9.993)*	4.363 (1.667–11.422)*
cAG or GG genotype with betel-nut chewing	51 (24.3%)	239 (61.6%)	18.745 (8.160–43.059)*	23.896 (8.838–64.610)*
**rs3829078**				
aAA genotype and no betel-nut chewing	130 (61.9%)	51 (13.1%)	1.00	1.00
bAG or GG genotype or betel-nut chewing	72 (34.3%)	303 (78.1%)	10.727 (7.095–16.219)*	15.182 (8.924–25.827)*
cAG or GG genotype with betel-nut chewing	8 (3.8%)	34 (8.8%)	10.833 (4.698–24.981)*	14.249 (5.183–39.173)*
**rs1048638**				
aCC genotype and no betel-nut chewing	118 (56.2%)	52 (13.4%)	1.00	1.00
bCA or AA genotype or betel-nut chewing	85 (40.5%)	298 (76.8%)	7.956 (5.303–11.935)*	10.652 (6.386–17.767)*
cCA or AA genotype with betel nut chewing	7 (3.3%)	38 (9.8%)	12.319 (5.163–29.393)*	16.798 (5.823–48.456)*
**376del393**				
aINS/INS genotype and no betel-nut chewing	107 (50.9%)	46 (11.9%)	1.00	1.00
bINS/Del or Del/Del genotype or betel-nut chewing	80 (38.1%)	259 (66.8%)	7.531 (4.914–11.541)*	11.301 (6.503–19.640)*
cINS/Del or Del/Del genotype with betel-nut chewing	23 (11.0%)	83 (21.4%)	8.394 (4.715–14.945)*	12.848 (6.188–26.677)*

The ORs with their 95% CIs were estimated by logistic regression models. The AORs with their 95% CIs were estimated by multiple logistic regression models after controlling for age, gender, and alcohol consumption.

a Individuals with the wild genotype but without betel-nut chewing.

b Individuals with either at least 1 mutated genotype or betel-nut chewing.

c Individuals with both at least 1 mutated genotype and betel-nut chewing.

* Statistically significant at *p*<0.05.

Among 444 betel-nut consumers in our cohort, tobacco smoking significantly elevated the oral cancer risk in participants polymorphic for the rs2071676, rs1048638, and 376del393 polymorphisms of *CA9*, compared to people with the WT gene who did not smoke cigarettes (Table 4). Moreover, people who were either polymorphic for *CA9* in 4 loci (+201, +1081, +1584, and +376) or who smoked were at a 4.13∼22.71-fold risk (*p*<0.001) of developing oral cancer, compared to people with the WT gene who did not smoke (Table 4). The above results suggest that *CA9* gene polymorphisms exert a strong influence on oral-cancer susceptibility who chews betel nut and/or smoke cigarettes.

**Table 4:** Adjusted odds ratios (ORs) (AORs) and 95% confidence interval (CIs) of oral cancer associated with *CA9* genotypic frequencies and smokers among 444 betel-nut consumers.

Variable	Controls (*n* = 91) (%)	Patients (*n* = 353) (%)	OR (95% CI)	AOR (95% CI)
**rs2071676**				
a AA genotype and no smoking	6 (6.6%)	4 (1.1%)	1.00	1.00
b AG or GG genotype or smoking	34 (37.4%)	110 (31.2%)	4.853 (1.293–18.209)*	13.177 (1.759–98.678)*
c AG or GG genotype with smoking	51 (56.0%)	239 (67.7%)	7.029 (1.914–25.813)*	18.568 (2.519–136.890)*
**rs3829078**				
a AA genotype and no smoking	17 (18.7%)	21 (5.9%)	1.00	1.00
b AG or GG genotype or smoking	66 (72.5%)	298 (84.4%)	3.655 (1.828–7.308)*	4.130 (1.682–10.139)*
c AG or GG genotype with smoking	8 (8.8%)	34 (9.6%)	3.440 (1.264–9.362)*	3.207 (0.917–11.217)*
**rs1048638**				
a CC genotype and non-smoker	17 (18.7%)	20 (5.7%)	1.00	1.00
b CA or AA genotype or smoking	67 (73.6%)	295 (83.6%)	3.743 (1.861–7.528)*	4.569 (1.830–11.406)*
c CA or AA genotype with smoking	7 (7.7%)	38 (10.8%)	4.614 (1.642–12.969)*	4.987 (1.400–17.762)*
**376del393**				
a INS/INS genotype and no smoking	17 (18.7%)	12 (3.4%)	1.00	1.00
b INS/Del or Del/Del genotype or smoking	51 (56.0%)	258 (73.1%)	7.167 (3.228–15.913)*	22.708 (6.682–77.169)*
c INS/Del or Del/Del genotype with smoking	23 (25.3%)	83 (23.5%)	5.112 (2.139–12.220)*	14.355 (3.944–52.247)*

The ORs with their 95% CIs were estimated by logistic regression models. The AORs with their 95% CIs were estimated by multiple logistic regression models after controlling for age, gender, and alcohol consumption.

a Individuals with the wild-type genotype but without smoking.

b Individuals with either at least 1 mutated genotype or smoking.

c Individuals with both at least 1 mutated genotype and smoking.

* Statistically significant at *p*<0.05.

We further explored the haplotypes to evaluate the combined effects of the 3 polymorphisms on oral-cancer susceptibility. The distribution frequencies of the *CA9* rs2071676, rs3829078, and rs1048638 haplotypes in our recruited individuals were analyzed. The most common haplotype in the control was AAC (46.6%), and it was therefore chosen as a reference. Compared to the reference, 1 *CA9* haplotype, GAA, significantly (*p*<0.001) increased the risks for OSCC by 5.27-fold (95% CI: 1.13–24.55) (Table 5).

**Table 5:** Distribution frequency of *CA9* haplotypes in controls and oral-cancer patients.

Variable			Controls (*N* = 1038) *n* (%)	Patients (*N* = 924) *n* (%)	OR (95% CI)	*p* value
rs2071676A/G	rs3829078A/G	rs1048638C/A				
A	A	C	484 (46.6%)	413 (44.7%)	Reference	
G	A	C	451 (43.4%)	404 (43.8%)	1.050 (0.870–1.267)	0.612
A	A	A	66 (6.4%)	53 (5.7%)	0.941 (0.641–1.382)	0.757
G	G	C	35 (3.4%)	41 (4.4%)	1.373 (0.858–2.196)	0.858
G	A	A	2 (0.2%)	9 (1.0%)	5.274 (1.133–24.545)	0.018*
A	G	C	0 (0%)	4 (0.4%)	**–**	**–**

OR, odds ratio; CI, confidence interval.

To explore the effects of polymorphic genotypes of *CA9* on the clinical status of OSCC, we classified OSCC patients into 2 subgroups. In the first subgroup, patients had homozygous WT alleles; in the other subgroup they had at least 1 polymorphic allele. For the genotypic frequencies of the SNPs, only *CA9* rs2071676 showed a significant association with clinical pathological variables in OSCC patients. Compared to the WT genotype (A/A), patients with at least 1 polymorphic G allele of rs2071676 showed a higher risk (1.69-fold; 95% CI: 1.00–2.84) for lymph node metastasis (Table 6).

**Table 6:** Distribution frequency of the clinical status and *CA9* rs2071676 genotype frequencies in 462 patients with oral cancer.

	Genotypic frequencies			
Variable	AA (*N* = 116)*n* (%)	AG+ GG (*N* = 346) *n* (%)	OR (95% CI)	AOR (95% CI)
Clinical Stage				
Stage I/II	59 (50.9%)	154 (44.5%)	1.00	1.00
Stage III/IV	57 (49.1%)	192 (55.5%)	1.290 (0.847–1.967)	1.240 (0.774–1.987)
Tumor size				
≤ T2	75 (64.7%)	218 (63.0%)	1.00	1.00
> T2	41 (35.3%)	128 (37.0%)	1.074 (0.693–1.666)	0.986 (0.601–1.615)
Lymph node metastasis				
No	85 (73.3%)	213 (61.6%)	1.00	1.00
Yes	31 (26.7%)	133 (38.4%)	1.712 (1.076–2.725)*	1.686 (1.002–2.839)*
Distant metastasis				
No	116 (100%)	338 (97.7%)	1.00	1.00
Yes	0 (0%)	8 (2.3%)	–	–
Cell differentiation				
Well	20 (17.2%)	48 (13.9%)	1.00	1.00
Moderately or poorly	96 (82.8%)	298 (86.1%)	1.293 (0.731–2.287)	1.328 (0.707–2.496)

The odds ratios (ORs) with 95% confidence intervals (CIs) were estimated by logistic regression models. The adjusted ORs (AORs) with 95% CIs were estimated by multiple logistic regression models after controlling for age, gender, betel-quid chewing, alcohol consumption, and tobacco use.

> T2: tumor size >2 cm in greatest dimension.

* Statistically significant at *p*<0.05.

## Discussion

Compared to the control group, a higher proportion of oral-cancer patients used betel quid, tobacco, or alcohol (Table 1). This finding suggests that these 3 environmental carcinogens may be associated with risks for oral cancer. Betel-quid and tobacco consumption appeared to be particularly strongly associated with oral cancer, a finding that is congruent with prior research [20]. Betel-quid chewing was found to stimulate the protein level of matrix metalloproteinase (MMP)-9 in the saliva of healthy people [21]. Lime-piper betel quid may also increase protein levels of c-fos and c-jun proto-oncogenes [22]. Consumption of tobacco may significantly induce the expression of nuclear hypoxia-inducible factor (HIF)-1α, which is an unfavorable prognostic factor in oral cancer [23]. These lines of evidence suggest that environmental carcinogen exposure is involved in the formation or pathogenesis of oral cancer.

**Table 7:** TaqMan primer sets for *CA9* genotyped SNPs.

SNP	Primer sequences	Probe
*CA9*+201 rs2071676	5′- GTGCAACTGCTGCTGTCAC -3′ 5′- CAACCTATGGGGATGGACA -3′	VIC-5′-CTGCTTCTGATGCCTGTCCFAM-5′-CTGCTTCTGGTGCCTGTCC
*CA9*+1081 rs3829078	5′-CTCCTGCCCTCTGACTTCAG-3′ 5′-AGGGCGGTGTAGTCAGAGA-3′	VIC-5′-CGCTACTTCCAATATGAFAM-5′-CGCTACTTCCGATATGA
*CA9*+1584 rs1048638	5′-TGTCCTGCTCATTATGCCACTTC-3′ 5′-GGGAACAAAGGTGACTAACACATATTT-3′	VIC-5′-CTTTTAACTGCAAAGAAATFAM-5′-TTTTAACTGCCAAGAAAT
*CA9*+376 Del	5′-TCTGCCAGTGAAGAGGATT-3′ 5′-TCTCCAGGAGCCTCAACAGT-3′	

CA9 expression is highly sensitive to hypoxic conditions [24]. The aberrant expression of CA9 was observed in cancer cells themselves or tumor-associated stromal tissues in OSCC patients and correlated with a poor prognosis and nodal metastasis [17], [18], [25]. CA9 catalyzes the reversible hydration of carbon dioxide to carbonic acid. This leads to intracellular alkalosis and extracellular acidosis in the tumor microenvironment, which allows tumors to survive under hypoxic conditions. Furthermore, extracellular acidosis may contribute to the invasiveness and poor prognosis of high-stromal CA9-expressing tumors by activating extracellular proteases and disrupting cell adhesion molecule function [26]–[28]. CA9 reduces E-cadherin-mediated cell adhesion by a competitive interaction with beta-catenin [28]; this may enhance the metastatic phenotype and contribute to the association between high-stromal CA9 expression and lymph-node metastases. Thus, CA9 expression by tumors may indicate the presence of hypoxic cells with more-aggressive behavior and treatment resistance due to hypoxia-induced cellular changes [29], [30].

Increasing evidence indicates that genomic changes progressively alter the cellular phenotype and might lead cells to evolve from a preneoplastic stage into cancer more readily [31]. It was reported that the oral mucosa of individuals with the *murine double minute 2 (MDM2)* SNP 309 GG genotype is more susceptible to environmental carcinogen exposure and results in an earlier onset of tumor formation [32]. The longer allelic polymorphism of the GT dinucleotide in the *heme oxygenase (HO)-1* promoter and functional polymorphism in the *nuclear factor kappa B1 (NFKB1)* promoter are both related to the risks of betel-quid- or smoking-related OSCC [5], [33]. Polymorphisms of several genes were identified as being associated with risks of oral cancer [34], [35]. It is clear that genetic components play pivotal roles in carcinogenesis. In our study, *CA9* gene SNPs (rs2071676, rs3829078, rs1048638, and 376del393) alone did not contribute to oral-cancer susceptibility (Table 2). The synergistic effect of environmental factor (betel quid and smoking) and *CA9* gene polymorphisms on the risk of oral cancer (Tables 3 and 4) are well demonstrated. Betel-quid and tobacco carcinogens might enhance HIF-1α expression, and then alter *CA9* promoter activity and upregulate CA9 expression. Consequently, it may upregulate extracellular proteases or downregulate E-cadherin to promote development of oral cancer.

It was reported that high expression of CA9 was more frequent with lymph-node metastasis in several kinds of solid tumors including oral cancer [25], [36], [37]. In the present study, a significantly higher distribution frequency of lymph-node metastasis was exhibited in OSCC patients with at least 1 polymorphic G allele of *CA9*+201 compared to those with the WT genotype (Table 6). The +201 A/G polymorphism is known to cause a nonsynonymous substitution (valine to methionine), but the expression and function of CA9 which are affected by this SNP are still unconfirmed. Although a study by de Martino et al. showed that this nonsynonymous SNP had no significant effect on CA9 protein expression [11], we think that the sample size in de Martino’s study (*n* = 54) was too small to determine whether the *CA9*+201 SNP had a significant effect on CA9’s expression and must be confirmed in larger prospective studies.

A variety of SNPs might be silent, that is to say, with no direct effect on gene products. However, by virtue of linkage disequilibrium (LD) that exists across the human genome, they can still be used as genetic markers to locate adjacent functional variants that contribute to disease. When each SNP constructing the haplotypes has a true contribution to the susceptibility of disease, even though unapparent, haplotype analyses can provide greater statistical power and are sometimes advantageous over analysis of individual SNPs for detecting an association between alleles and a disease phenotype [38]. We analyzed contributions of different haplotype combinations of 3 *CA9* SNPs (rs2071676, rs3829078, and rs1048638) to the risk of oral cancer and eventually found that the *GAA* haplotype showed a high risk for OSCC (Table 5). It is possible that the *GAA* haplotype of *CA9* is in LD with other functional polymorphisms that are responsible for the susceptibility to OSCC.

Interpretations of this study are limited because information on certain oral-cancer risk factors, such as marijuana (cannabis) smoking, medicinal nicotine use, heredity, and familial risks, were not available for the recruited specimens, and this limitation may restrict the adjustment of these possibly confounding factors. In this study, however, the major risk factors for oral cancer, of alcohol and tobacco consumption and betel-quid chewing, were adjusted for in order to estimate the effects of gene polymorphisms on the clinicopathological development of OSCC. Some indirect adjustments, such as for age, gender, and geographic area, were also used to evaluate the effects of gene polymorphisms on oral cancer between healthy control subjects and OSCC patients to reduce the possibility of false-positive results. In a future study, increasing the specimen (blood and tumor tissue) number and taking more OSCC risk factors into account in the analysis might precisely validate these findings.

In conclusion, our results suggest that gene-environment interactions between *CA9* polymorphisms and betel-quid chewing with smoking may alter the susceptibility to oral-cancer development. The *GAA* haplotype of the 3 *CA-9* SNPs (rs2071676, rs3829078, and rs1048638) combined also showed a high risk association with OSCC. Oral-cancer patients with the *CA9*+201 A/G polymorphism have a higher risk of having neck lymph-node metastasis than do WT carriers.

## Materials and Methods

### Subjects and Specimen Collection

We recruited 462 patients (444 men and 18 women, with a mean age of 54.4±11.4 years) at Chung Shan Medical University Hospital in Taichung, and Changhua Christian Hospital and Show Chwan Memorial Hospital in Changhua, Taiwan. Patients were enrolled as a case group in 2007–2010. Among the 462 cases, tumors were located in the buccal mucosa (n  = 189), tongue (n  = 148), gingiva (n  = 48), palate (n  = 30), floor of the mouth (n = 21), and others (n  = 26). Meanwhile, controls were enrolled from the physical examination during those three hospitals, which are also the facilities that cases were collected from. At the end of recruitment, a total of 519 participants (425 men and 94 women, with a mean age of 52.4±14.7 years) that had neither self-reported history of cancer of any sites were included. In addition, subjects with oral precancerous disease such as oral submucous fibrosis, leukoplakia, erythroplakia, verrucous hyperplasia, etc. were excluded from control group. Before commencement of this study, approval was obtained from the Institutional Review Board of Show Chwan Memorial Hospital, and informed written consent to participate in the study was obtained from each person.

For both cases and controls, we used a questionnaire to obtain information on patient exposure to betel-quid chewing, tobacco use, and alcohol consumption. Medical information for the cases was obtained from their medical records, and included TNM clinical staging, primary tumor size, lymph node involvement, and histologic grade. Oral-cancer patients were clinically staged at the time of diagnosis according to the TNM staging system of the American Joint Committee on Cancer (AJCC) Staging Manual (7th ed.) [39]. Tumors are classified as stage I and stage II (n  = 213) and stage III+stage IV (n  = 249). Metastasis into lymph nodes was detected in 164 cases (35.5%). Whole-blood specimens collected from controls and OSCC patients were placed in tubes containing ethylenediaminetetraacetic acid (EDTA), immediately centrifuged, and stored at −80°C.

### Selection of CA9 Polymorphisms

In dbSNP database, over 30 SNPs has been documented in the 11 exons region of the *CA9* gene, including three SNPs located in the coding sequences of the gene (Exons 1, 7 and 11). To obtain adequate power for evaluating the potential association, we investigated rs2071676 (G201A in exon 1), rs3829078 (A1018G in exon 7) and rs1048638 (C1584A in 3′UTR), those with minor allele frequencies ≥5%. Furthermore, another SNP of *CA9* gene (an 18-bp deletion/insertion; +376 Del) was selected in this study since this SNP was found in the cancer patients [11].

### Genomic DNA Extraction

Genomic DNA was extracted using QIAamp DNA blood mini kits (Qiagen, Valencia, CA, USA) following the manufacturer’s instructions. We dissolved DNA in TE buffer (10 mM Tris and 1 mM EDTA; pH 7.8) and then quantified it by measuring the OD260. The final preparation was stored at -20°C and was used to act as templates for the polymerase chain reaction (PCR).

### Real-time PCR

Allelic discrimination of the *CA9*+201 (rs2071676), +1081 (rs3829078) and +1584 (rs1048638) allelic polymorphisms were assessed with an ABI StepOne™ Real-Time PCR System (Applied Biosystems, Foster City, CA, USA), and analyzed with SDS vers. 3.0 software (Applied Biosystems) using the TaqMan assay. Furthermore, the 376del393 allelic polymorphisms were assessed with PCR. The products were separated on a 3% agarose gel and then stained with ethidium bromide. The primer sequences and probes for analysis of the *CA9* gene polymorphisms are described in Table 7. The final volume for each reaction was 5 µL, containing 2.5 µL TaqMan Genotyping Master Mix, 0.125 µL TaqMan probe mix, and 10 ng genomic DNA. In our preliminary study, such reaction mixture (5 µL) has been proven to provide identical results to that of with a final volume of 20 µL recommended in the manufacturer’s instruction. The real-time PCR included an initial denaturation step at 95°C for 10 min, followed by 40 cycles at of 95°C for 15 s and then at 60°C for 1 min. To validate results from real time PCR and PCR, around 10% of assays were repeated and several cases of each genotype were confirmed by the DNA sequence analysis.

### Statistical Analysis

Differences between the 2 groups were considered significant if *p* values were <0.05. Hardy-Weinberg equilibrium (HWE) was assessed using a goodness-of-fit *Χ^2^*-test for biallelic markers. The Mann-Whitney *U*-test and Fisher’s exact test were used to compare differences in distributions of patient demographic characteristics between the cancer-free (control) and oral-cancer groups. The adjusted odds ratios (AORs) and 95% confidence intervals (CIs) of the association between genotype frequencies and risk plus clinicopathological characteristics were estimated using multiple logistic regression models, after controlling for other covariates. We analyzed all data with Statistical Analytic System (SAS Institute, Cary, NC, USA) software (vers. 9.1, 2005) for Windows.
